# Effect of different levels of neutral detergent fiber in starter diets on the performance, ruminal fermentation, and structural growth of Holstein calves

**DOI:** 10.3389/fvets.2025.1557732

**Published:** 2025-04-30

**Authors:** Chunyan Ren, Yanliang Bi, Yan Tu, Yanli Guo, Rui Bing, Jiqing Wang, Qiyu Diao

**Affiliations:** ^1^College of Animal Science and Technology, Gansu Agricultural University, Lanzhou, China; ^2^Key Laboratory of Feed Biotechnology of the Ministry of Agriculture, Feed Research Institute, Chinese Academy of Agricultural Sciences, Beijing, China; ^3^Longnan Animal Disease Control Center, Longnan, China

**Keywords:** starter, forage, NDF, performance, ruminal fermentation, calves

## Abstract

We evaluated the effect of starter diets with four levels [12.85, 19.91, 26.99, and 34.04% of dry matter (DM)] of neutral detergent fiber (NDF) on the growth, ruminal fermentation, and structural growth of Holstein calves. A total of 60 Holstein calves [42.3 ± 1.1 kg, body weight (BW)] aged 1–3 days were randomly assigned to 4 treatments: a starter diet with a dry matter basis without hay forage, 12.85% NDF; a starter diet with 14% forage, 19.91% NDF; a starter diet with 28% forage, 26.99% NDF; and a starter diet with 42% forage, 34.04% NDF. The hay forage (alfalfa hay and oat grass) was pelleted together with the concentrate to provide the calves, which had *ad libitum* access to water and starter feed throughout the experiment. Following weaning at 70 days, the calves continued on their respective diets until the trial termination at 112 days of age. The average daily gain (ADG) decreased linearly (*p* = 0.02) with increasing dietary NDF concentration. The starter intake and total dry matter intake (TDMI) increased quadratically (*p* = 0.02 and *p* = 0.03, respectively) with increasing NDF concentration in the starter diets, with maximal values observed at 26.99% NDF. Feed efficiency (FE) exhibited a decreasing trend with elevated NDF levels in the starter formulations during the post-weaning period, whereas diets containing 12.85 and 19.91% NDF diets maintained superior efficiency. Notably, the NDF intake showed a linear increase (*p* < 0.01) during both the pre- and post-weaning periods as the starter NDF levels escalated. The pH value exhibited either a linear or quadratic relationship with increasing dietary NDF levels at 112 days of age. The valerate concentrations showed a linear decreasing trend with increasing dietary NDF levels at 112 days of age. The total volatile fatty acid (TVFA) concentrations increased linearly with increasing dietary NDF levels. Elevating dietary NDF levels showed a linear reduction in the final body weight (BW) and weaning BW. The calf body length exhibited a linear decrease with increasing NDF levels, with the 12.85 and 19.91% NDF diets yielding greater values at 112 days of age. The blood beta-hydroxybutyric acid (BHBA) concentration was linearly elevated by higher dietary NDF levels at 70 days of age. Under the experimental conditions, the dietary NDF level of 12.85% or 19.91% enhanced the average daily gain (ADG), BW, and structural growth parameters. These findings indicate that the dietary NDF content below 26.99% constitutes the optimal range for Holstein calves aged 1–3 months.

## Introduction

Traditionally, in an attempt to accelerate weaning, reduce the risk of scours, and lower the costs of feeding and management, weaning strategies have aimed to limit the amount of milk or milk replacers supplied to calves and to encourage early grain consumption (1). Based on alterations in microbial digestive and metabolic products, the composition of the calf starter diet plays a crucial role in the anatomical and functional development of the rumen (2–4). Feeding concentrate diets to young calves during the pre- and post-weaning periods might decrease rumen pH (5), cause hyperkeratinization, and lead to rumen papillae clumping and branching (6), which can impair rumen mucosa absorption (7), especially when calves are fed starter diets with a fine particle size (8). Various reports indicate that feeding forage before weaning is generally discouraged, as forage is less energy-dense compared to concentrated diets, leading to lower body weight (BW) gains in calves (1, 9, 10). In addition, forage leads to excessive acetate production, which delays the development of rumen papillae and inhibits the growth of young ruminants (11–13). However, many studies have demonstrated that adding forage to a young calf’s starter diet can improve feed intake, average daily gain (ADG), and growth performance and promote ruminal fermentation and development (14–17). This process is triggered by the muscular layer of the rumen (12) and an increase in the rumen’s buffering capacity (14).

Evidence shows that the forage level is one of the most critical elements influencing the growth performance of dairy calves (18). In a previous study, Porter et al. (19) observed that calves fed high-fiber starters (27% neutral detergent fiber (NDF)) had numerically greater body weight gain and starter intake compared to those fed low-fiber starters (20% NDF) during the pre-weaning period. Terré et al. (20) reported that the ADG was greater with low-NDF starters (18% NDF) than with high-NDF (27% NDF) starters. Nemati et al. (21) demonstrated that providing calves with a diet containing 25% alfalfa hay (24.5% NDF) can increase DMI and weight gain in dairy calves compared to a diet containing 12.5% alfalfa hay (23% NDF). However, there is limited information on how dietary NDF levels affect growth performance, and the optimal NDF level during the pre- and post-weaning stages in dairy calf diets is still unknown. Therefore, determining the optimal level of NDF for starter diets to improve production performance and promote ruminal development will benefit dairy farmers. We hypothesized that the optimal NDF level in diets would improve the ADG, starter intake, feed efficiency, ruminal fermentation, and body growth in calves. Thus, the present study aimed to determine the optimal dietary NDF level for Holstein dairy calves.

## Materials and methods

### Animal management and treatment

The study protocol received approval from the Animal Ethics Committee of the Chinese Academy of Agriculture Science (Beijing, China).

This research was carried out on a commercial dairy farm Yin Xiang Wei Ye Co. in Shandong Province, China from April to August 2017. A total of 60 newborn Holstein calves (36 bulls and 24 heifer calves) with a BW value of 42.3 ± 1.1 kg were randomly assigned to four treatments (nine male and six female calves per treatment). The treatments included the following: a pelleted starter without forage (12.85% NDF) and pelleted starters supplemented with 14% forage (19.91% NDF), 28% forage (26.99% NDF), and 42% forage (34.04% NDF). Alfalfa hay and oat grass were pelleted together with the concentrate for feeding. The calves were separated from their mothers immediately after birth, weighed, and housed individually in pens (3.4 m length × 1.5 m width × 1.5 m height) with sand bedding. Each calf received 4 L of colostrum within the first 2 h after birth.

Whole milk samples were collected every 2 weeks to determine crude protein, fat, and total solids using an infrared spectrophotometer (Foss MilkoScan, Foss Electric, Hillerod, Denmark). The average milk composition was 3.11 ± 0.07% (mean ± SD) crude protein, 3.80 ± 0.08% fat, 4.88 ± 0.05% lactose, and 8.87% total solids. The calves were fed whole milk twice daily at 06:30 h and 17:00 h using a bucket, starting from 2 days old. The feeding rates were 5 L/day for 2–28 days old, 8 L/day for 29–65 days old, and 4 L/day for 66–70 days old. The calves were weaned at 70 days old, and the study concluded at 112 days old. The calf starter was offered at 15 days old. The calf starter and fresh water were offered *ad libitum*.

Alfalfa hay and oat grass were processed using a 9FQ-42 hammer mill (Qingdao Shandong, China) to achieve a particle size of 6 mm. The forage and other compositions were mixed, pelleted, and formulated to meet the National Research Council (NRC) (2001) requirements, maintaining a crude protein content of 18.8%. Table 1 details the composition and nutrient levels of the starter diets.

**Table 1:** Composition and nutrients of the experimental starter feeds (%, DM basis).

Item	Treatment^3^			
	12.85% NDF	19.91% NDF	26.99% NDF	34.04% NDF
Ingredients, %				
Corn	60	48.7	38	26.5
Soybean meal^4^	27.7	25.3	22.2	20.13
Wheat bran^5^	8	8	8	8
Alfalfa hay	0	6.9	16	21.8
Oat grass	0	7	12	20
Limestone	2.22	2	1.67	1.45
CaHPO_4_	0.73	0.75	0.78	0.77
Salt	0.35	0.35	0.35	0.35
Premix^1^	1	1	1	1
Total	100	100	100	100
Nutrient compositions				
Dry matter, %	88.30	86.83	88.72	88.69
ME, MJ/kg^2^	2.73	2.61	2.48	2.36
Crude protein, % of DM	18.40	18.46	18.87	18.97
Ether extract, % of DM	4.60	3.28	3.21	3.50
Neutral detergent fiber, % of DM	12.85	19.91	26.99	34.04
Acid detergent fiber, % of DM	6.30	10.50	12.75	16.42
Ash, % of DM	7.58	7.77	8.59	9.19
Ca, % of DM	1.15	1.09	1.15	1.10
P, % of DM	0.50	0.49	0.53	0.48

1 1 kg of premix supplied of the starter diets: VA 15,000 IU, VD 5,000 IU, VE 50 mg, VB 52 mg, Fe 90 mg, Cu 12.5 mg, Mn 30 mg, Zn 90 mg, P 20 mg, Mg 20 mg, Na 18 mg, S 30 mg, Se 0.3 mg, I 6.0 mg, and Co 0.5 mg.

2 ME was calculated, while others were measured values.

3 Treatments: 12.85% NDF = calf starter containing 12.85% neutral detergent fiber (NDF) on a dry matter (DM) basis; 19.91% NDF = calf starter containing 19.91% NDF on a DM basis; 26.99% NDF = calf starter containing 26.99% NDF on a DM basis; and 34.04% NDF = calf starter containing 34.04% NDF on a DM basis.

4 Alfalfa hay, DM 92.56%, EE 1.71%, CP 19.61%, NDF 42.66%, ADF 28.10%, Ash11.08%.

5 Oat hay DM 88%, EE 1.88%, CP 7.74%, NDF 49.3%, ADF 30.98%, Ash 7.78%.

### Sample collection and analysis

Throughout the experiment, the starter intake was recorded daily, and the BW was recorded every 2 weeks. Means of the total dry matter intake (TDMI), including milk solids and starter, average daily gain (ADG), and feed efficiency (FE), were calculated in the periods of 15–70 days and 71–112 days of age.

Starter and forage samples were obtained every week throughout the study and stored at −20°C for subsequent chemical analysis. The dry matter (DM), crude protein (CP), calcium (Ca), phosphorus (P), ether extract, and ash contents were analyzed according to the methods of AOAC (22). The NDF and ADF contents were analyzed using an ANKOM fiber analyzer (A2000i; American ANKOM, Macedon, NY, United States), with heat-stable alpha-amylase added for NDF analysis (23).

Two hours post-morning feed, ruminal samples (30 mL) were collected from 6 bull calves per treatment using a stomach tube at 70 and 112 days of age. The initial 50 mL of the ruminal fluid was discarded to avoid contamination from previous animals or the calves’ own saliva. Subsequently, 30 mL of the ruminal fluid was collected from each calf, and its pH was immediately measured using a pH meter (pH Testr 30; Oakton Instruments, Vernon Hills, IL, United States). Then, 10 mL of the ruminal fluid was acidified with 3 mL of 34.04% metaphosphoric acid and kept at −20°C until further examination for volatile fatty acids (VFAs). VFAs were measured as described in the study of Yang and Varga (24). Briefly, 5 mL of a rumen fluid filtrate was added to 1 mL of a metaphosphoric acid solution (250 g/L) and 1 mL of 0.6% 2-methyl-butyrate as an internal standard, mixed overnight, and analyzed via gas chromatography (GC522, Wufeng Instruments, Shanghai, China).

At 70 and 112 days of age, the calves were measured for body length (distance between the points of the shoulder and rump), withers height (distance from the base of the front feet to the withers), heart girth (circumference of the chest), hip height (distance from the base of the rear feet to the hook bones), and hip width (distance between the points of the hip bones), following the methodology of Khan et al. (4).

Blood samples were collected from the jugular vein into vacuum tubes 1 h before the morning feed on days 70 and 112 and then immediately placed on ice. The samples were centrifuged for 10 min at 3,000 × g (at 4°C) within 2 h of collection. The serum was extracted and stored at −20°C for further analysis. Glucose (Glu) and blood urea nitrogen (BUN) concentrations were determined spectrophotometrically (Hitachi-7180; Hitachi, Tokyo, Japan) and evaluated using a commercial assay kit (Diasys Diagnostic Systems, Shanghai Co., Ltd., China) according to the manufacturer’s instructions. The beta-hydroxybutyric acid (BHBA) concentration was determined using an enzyme-linked immunosorbent assay (ELISA) kit (Nanjing Jiancheng Bioengineering Institute, Nanjing, Jiangsu, China).

### Statistical analysis

Data on the growth performance (days 15–70 and 71–112) and structural growth (days 70 and 112) were analyzed using a randomized complete block design via the PROC MIXED procedure in SAS (version 9.1, SAS Inst. Inc., Cary, NC, United States). The model employed was as follows:


$$y_{ijk}=\mu +n_{i}+s_{j}+t_{k}+{\left(n\times s\right)}_{ij}+{\left(n\times t\right)}_{ik}+e_{ijk}\text{,}$$

where *μ* is the overall mean, $y_{ijk}$ represents the phenotype in growth performance and structural growth, $n_{i}$ is the NDF level fixed effect levels (four levels: 12.85, 19.91, 26.99, and 34.04%), $s_{j}$ is the sex fixed effect (two levels: male, female), $t_{k}$ is the time fixed effect (two levels:15–70 days, 71–112 days), (*n* × *s*)*_ij_* represents the interaction between treatment and sex, (*n* × *t*)*_ik_* represents the interaction between treatment and day, and $e_{ijk}$ is the error term. The effect of the calves’ sex on growth performance and structural growth was not significant.

Ruminal fermentation (days 70 and 112) and blood metabolite data (days 70 and 112) variables were analyzed using a model excluding sex effects. The dose-dependent effects of the NDF level were assessed through linear and quadratic *p*-values using orthogonal contrasts. Each variable was analyzed using the following covariance structures: unstructured, compound symmetry, and autoregressive order 1. The covariance structure that yielded the minimum Bayesian information criterion was chosen. The results were presented as least squares means for each group, the standard error of the mean, and *p*-values for NDF level effects. Mean differences were deemed significant at a *p*-value of ≤0.05, with trends noted at 0.05 < *p* ≤ 0.10.

## Results

### Growth performance

The ADG decreased linearly (*p* = 0.02) with increasing dietary NDF concentration, with higher values observed at 12.85 and 19.91% NDF concentrations during the pre- and post-weaning periods, respectively (Table 2). During the pre-weaning period, starter intake and TDMI increased quadratically (*p* = 0.02 and *p* = 0.03, respectively) with increasing NDF concentration in the starter diets, with maximal values (*p* < 0.05) observed at the 26.99% NDF concentration. Notably, the NDF intake showed a linear increase (*p* < 0.01) during both pre- and post-weaning periods as the starter NDF levels increased. The FE exhibited a decreasing trend with elevated NDF levels in the starter formulations during the post-weaning period, with the 12.85 and 19.91% NDF diets maintaining superior efficiency (see Table 2).

**Table 2:** Effects of the starter NDF levels on the growth performance of the dairy calves.

Item	Treatment^1^					*p*-value^3^	
	12.85% NDF	19.91% NDF	26.99% NDF	34.04% NDF	SE	*L*	*Q*
No. of calves	11	14	12	12			
Pre-weaning (15–70 days)							
ADG, kg/day	0.76^a^	0.75^a^	0.740^ab^	0.73^b^	0.04	0.02	0.13
Starter intake, kg/day	0.44^b^	0.46^ab^	0.48^a^	0.45^b^	0.04	0.07	0.02
Total DMI, kg/day	1.24^b^	1.29^ab^	1.32^a^	1.28^b^	0.05	0.13	0.07
NDF intake, kg/day	0.05^c^	0.09^b^	0.13^a^	0.11^a^	0.01	0.69	0.02
Feed efficiency^2^	0.65	0.64	0.62	0.62	0.04	0.24	0.12
Post-weaning (71–112 days)							
ADG, kg/day	0.84^a^	0.84^a^	0.72^b^	0.71^b^	0.04	<0.01	0.86
Total DMI, kg/day	2.37	2.61	2.53	2.75	0.17	0.78	0.13
Starter intake, kg/day	2.4	2.64	2.56	2.78	0.17	0.79	0.14
NDF intake, kg/day	0.31^d^	0.52^c^	0.69^b^	0.86^a^	0.04	<0.01	0.13
Feed efficiency^2^	0.40^a^	0.38^a^	0.28^b^	0.27^b^	0.03	0.09	0.22

1 Treatment: 12.85% NDF = calf starter containing 12.85% neutral detergent fiber (NDF) on a dry matter (DM) basis; 19.91% NDF = calf starter containing 19.91% NDF on a DM basis; 26.99 %NDF = calf starter containing 26.99% NDF on a DM basis; and 34.04% NDF = calf starter containing 34.04% NDF on a DM basis.

2 Feed efficiency was calculated by dividing the average daily gain (ADG) (g) by the daily total dry matter intake (TDMI).

3 *p*-value: L = linear, Q = quadratic effect of starter NDF contents (12.85, 19.91, 26.99 vs. 34.04% NDF, DM basis).

### Ruminal fermentation

The pH value exhibited either a linear or quadratic relationship with increasing dietary NDF levels (Table 3). The valerate concentrations showed a linear decreasing trend with increasing dietary NDF levels at 112 days of age. The total volatile fatty acid (TVFA) concentrations increased linearly with increasing dietary NDF levels. No differences were observed in the molar proportion of the acetate, propionate, butyrate, valerate, and total VFA concentrations at 70 days of age.

**Table 3:** Effects of the starter NDF levels on pH and VFA concentrations in the rumen fluid of the dairy calves.

Item	Treatment^1^					*P*-value^3^	
	12.85%NDF	19.91%NDF	26.99% NDF	34.04%NDF	SE	*L*	*Q*
No. of calves	6	6	6	6			
pH							
70 days	5.51	5.39	5.7	5.56	0.05	0.05	0.79
112days	6.21^a^	6.25^a^	5.55^b^	5.82^b^	0.08	0.01	< 0.01
Acetate (A), mol /100 mol							
70 days	48.06	51.64	54.71	53.19	1.23	0.12	0.30
112 days	45.80	50.86	45.50	49.86	0.95	0.38	0.84
Propionate (P), mol /100 mol							
70 days	31.97	33.51	31.68	26.28	1.41	0.17	0.24
112 days	31.72	28.93	38.42	32.42	1.23	0.24	0.46
Butyrate, mol /100 mol							
70 days	12.11	9.98	10.02	13.42	1.16	0.72	0.27
112 days	10.9	9.32	10.6	12.36	0.59	0.29	0.17
Valerate, mol /100 mol							
70 days	4.94	3.01	2.24	1.92	0.55	0.07	0.46
112 days	3.91^a^	2.76^b^	2.46^c^	2.29^d^	0.24	0.01	0.27
A/P ratio^2^							
70 days	1.56	1.6	1.78	2.04	0.08	0.05	0.51
112 days	1.49	1.79	1.21	1.65	0.09	0.90	0.68
Total VFA, mmol/L							
70 days	136.8	126.14	94.5	126.22	7.27	0.34	0.15
112 days	106.27^b^	101.14^b^	145.01^a^	155.06^a^	7.31	< 0.01	0.52

^1^ Treatment: 12.85% NDF = calf starter containing 12.85% neutral detergent fiber (NDF) on a dry matter (DM) basis; 19.91% NDF = calf starter containing 19.91% NDF on a DM basis; 26.99 %NDF = calf starter containing 26.99% NDF on a DM basis; 34.04% NDF = calf starter containing 34.04% NDF on a DM basis.

^2 A/P = the acetate-to-propionate ratio^

^3^ *P*-value: L = linear and Q = quadratic effect of starter NDF contents (12.85, 19.91, 26.99 *vs*. 34.04% NDF, DM basis).

### Structural growth

The initial weights of the calves were similar (*p* > 0.05, Table 4). Elevating dietary NDF levels showed a linear decrease in the final body weight (BW) (*p* = 0.04) and weaning BW (*p* = 0.05). The calves’ body length exhibited a linear decrease (*p* = 0.04) with increasing NDF levels, with the 12.85 and 19.91% NDF diets yielding greater values at 112 days of age. No interaction was observed between the NDF level and age for wither heights, hip height, hip width, or heart girth at 70 and 112 days. However, all parameters increased with age.

**Table 4:** Effects of the starter NDF levels on the structural growth of the dairy calves.

Item	Treatment^1^					*p*-value^2^		
	12.85% NDF	19.91% NDF	26.99% NDF	34.04% NDF	SEM	*L*	*Q*	*D*
No. of calves	*n* = 12	*n* = 13	*n* = 13	*n* = 14				
Birth BW, kg	43.05	42.25	42.21	42.34	1.58	0.11	0.45	
Weaning BW, kg	87.84^a^	87.84^a^	87.39^a^	85.74^b^	2.93	0.05	0.32	
Final BW, kg	122.91^a^	124.38^a^	113.84^b^	114.92^b^	4.35	0.04	0.70	
Wither heights, cm								
70 days	89.90	87.36	85.86	86.59	1.49	0.32	0.79	
112 days	94.31	95.55	93.43	93.03	1.52	0.83	0.38	
Total	92.10	91.45	89.65	89.82	1.05	0.58	0.41	<0.01
Hip height, cm								
70 days	97.95	95.73	95.73	94.05	1.34	0.16	0.48	
112 days	102.02	103.17	101.09	101.07	1.70	0.54	0.96	
Total	99.99	99.44	97.22	97.57	1.07	0.17	0.62	<0.01
Hip width, cm								
70 days	24.50	24.63	25.24	24.54	0.53	0.76	0.81	
112 days	26.12	26.33	32.70	26.50	4.54	0.61	0.19	
Total	25.31	25.48	25.48	25.52	2.28	0.64	0.19	0.04
Heart girth, cm								
70 days	105.21	104.34	104.48	103.77	1.38	0.27	0.72	
112 days	119.13	120.10	117.70	117.15	2.04	0.14	0.81	
Total	112.17^a^	112.22^a^	111.09^ab^	110.46^b^	1.20	0.06	0.68	<0.01
Body length, cm								
70 days	93.91	88.84	91.78	91.06	4.53	0.85	0.86	
112 days	107.11^a^	107.97^a^	106.31^ab^	104.09^b^	2.26	0.04	0.13	
Total	100.51	98.40	99.04	97.56	2.55	0.25	0.56	<0.01

1 Treatment: 12.85% NDF = calf starter containing 12.85% neutral detergent fiber (NDF) on a dry matter (DM) basis; 19.91% NDF = calf starter containing 19.91% NDF on a DM basis; 26.99 %NDF = calf starter containing 26.99% NDF on a DM basis; and 34.04% NDF = calf starter containing 34.04% NDF on a DM basis.

2 *p*-value: L = linear and Q = quadratic effect of starter NDF contents (12.85, 19.91, 26.99 vs. 34.04% NDF, DM basis). D = day effect (days 70 and 112).

### Blood metabolites

The blood BHBA concentration was linearly elevated with higher dietary NDF levels at 70 days of age (*p* < 0.01; Table 5). In contrast, the blood glucose and BUN concentrations were unaffected by the NDF level (*p* > 0.05). Notably, the BUN concentration showed a significant age-dependent increase during the experimental period.

**Table 5:** Effects of the starter NDF levels on the structural growth of the dairy calves.

Item	Treatment^1^					*p*-value^2^			
	12.85% NDF	19.91% NDF	26.99% NDF	34.04% NDF	SEM	*L*	*Q*	*C*	*D*
No. of calves	*n* = 5	*n* = 5	*n* = 5	*n* = 5					
BHBA, mmol/L									
70 days	0.54^b^	0.54^b^	0.62^ab^	0.65^a^	0.04	<0.01	0.71	0.28	
112 days	0.62	0.58	0.6	0.58	0.02	0.19	0.56	0.32	
Total	0.57^b^	0.56^b^	0.61^a^	0.61^a^	0.02	0.04	0.53	0.14	0.67
Glu, mmol/L									
70 days	6.63	5.67	5.61	5.6	1.6	0.54	0.67	0.86	
112 days	6.42	4.33	5.05	4.31	1.14	0.13	0.39	0.21	
Total	6.52	5	5.33	4.95	0.97	0.16	0.38	0.36	0.2
BUN, mmol/L									
70 days	5.83	5.8	5.78	5.76	0.42	0.86	0.98	1	
112 days	6.7	6.4	6.48	6.71	0.33	0.91	0.26	0.82	
Total	6.27	6.1	6.13	6.24	0.26	0.94	0.45	0.88	<0.01

1 Treatment: 12.85% NDF = calf starter containing 12.85% neutral detergent fiber (NDF) on a dry matter (DM) basis; 19.91% NDF = calf starter containing 19.91% NDF on a DM basis; 26.99% NDF = calf starter containing 26.99% NDF on a DM basis; and 34.04% NDF = calf starter containing 34.04% NDF on a DM basis.

2 *p*-value: L = linear and Q = quadratic effect of starter NDF contents (12.85, 19.91, 26.99 vs. 34.04% NDF, DM basis). D = day effect (days 70 and 112).

## Discussion

### Growth performance

The present study showed that the ADG decreased linearly with increasing NDF concentration, with higher values observed in the 12.85 and 19.91% NDF diets during the pre-weaning and post-weaning periods, respectively. A linear function predicted peak ADG to occur at the 12.85% or 19.91% NDF dietary level, which agreed with the findings of a previous study (25). Optimal NDF and starch ratios are beneficial for demonstrating rumen environment-modulating effects (26), resulting in enhanced calf ADG. Other researchers have reported that forage supplementation in calf diets could significantly increase the ADG. This effect is attributed to the retention of forage within the gastric lumen and the increased weight of the gastrointestinal tissues (16, 20). In this study, increasing dietary NDF concentration had a negative effect on the ADG during the pre- and post-weaning periods, demonstrating that metabolic, rather than physical, factors regulate the high NDF levels in diet consumption (27). In the current study, we found that the rumen weight and kidney index were greater at the 12.85 and 19.91% NDF levels (28). Therefore, it is likely that the development of gastrointestinal tissues has a greater influence on the ADG than the retention of forage.

Previous studies have shown that the NDF level in starter diets affects DMI (4, 15, 18). The addition of roughage to the concentrate improves the ruminal environment and promotes the development of ruminal muscles, leading to increased DMI in calves. During the 3-week antepartum period, the DMI of non-lactating cows decreased as the content of roughage NDF in the diet was increased (29). Similar findings were reported for lactating cows in studies reviewed by West et al. (30). In the current study, the starter intake increased quadratically and the TDMI increased in a cubical manner, with no further increase observed between the 26.99 and 34.04% NDF concentrations during the pre-weaning period. These results indicated that increasing the starter NDF content beyond 26.99% did not result in benefits for the starter intake. A previous study noted that NDF intake was higher in calves fed high NDF diets compared to calves fed low NDF diets (20). In our study, the NDF intake aligned with the changes in the starter NDF content. As calves matured, higher starter intake and NDF utilization were associated with rumen volume expansion and enhanced solid fermentation (26, 31).

Previous studies (15, 16, 20, 21) have documented that forage supplementation does not enhance FE. In this study, the FE decreased with elevated NDF levels in the starter formulations during the post-weaning period, where the 12.85 and 19.91% NDF diets maintained superior efficiency. This finding aligns with other research (9, 10, 32), indicating that FE declines as NDF percentage increases, likely due to increased starter intake at higher NDF levels. These findings suggest that diets with NDF levels below 26.99% may improve ADG, FE, and DMI in calves.

### Ruminal fermentation

Previous studies (4, 14) have shown that forage supplementation in young calves elevates ruminal pH. Ruminal pH increased with increasing alfalfa content prior to weaning (16). Laarman et al. (33) indicated a positive relationship between hay intake and ruminal pH. In contrast, Suárez et al. (34) observed no change in ruminal pH when forage replaced part of the concentrate. The study found that the dietary NDF levels influenced pH values both linearly and quadratically, with the highest pH value observed at 12.85% or 19.91% NDF concentration at 112 days of age. This finding suggests that increased ruminal passage rates reduce fermentation time, decrease total VFA production, and raise pH. In addition, it might be linked to underdeveloped ruminal epithelium in calves, where volatile fatty acid production surpasses the absorptive capacity of the rumen wall, resulting in low ruminal pH (35).

A previous study found that a high-NDF diet (26.7% NDF) increased the acetate molar proportion in the rumen while decreasing the molar proportions of propionate, butyrate, and valerate (20). In contrast, Hill et al. (36) found no significant differences in VFA profiles among young dairy calves fed concentrates with varying NDF levels. Zitnan et al. (13) explained that higher-forage diets have a higher abundance of cellulolytic microorganisms, resulting in greater degradation of fiber and leading to increased acetate molar ratios in the rumen at 63 days old. However, this finding was inconsistent with the results of the current study. Previously, Cline et al. (37) reported that valerate utilization increases with microbial growth. In this study, the valerate concentrations showed a linear decreasing trend with increasing dietary NDF levels at both 70 and 112 days of age, which aligns with the findings of Khan et al. (4) and Terré et al. (20), who suggested that high-NDF diets might promote the growth of cellulolytic bacteria in calves’ rumens. Decreased ruminal pH hinders cellulolytic bacterial growth, resulting in a decline in the acetate-to-propionate ratio (38). In this study, the ruminal total VFA concentrations were within the range reported previously in the literature (18, 34, 39), showing a significant negative correlation with ruminal liquid pH. Ruminal fermentation, VFA build-up, and ruminal epithelial development in neonatal calves are closely associated with solid feed intake (starter) and NDF intake (31). The total VFA concentrations increased linearly with an increase in the NDF level at 112 days, which could have been caused by increased starter intake, NDF consumption, and the earlier establishment of rumen microorganisms post-weaning (17).

### Structural growth

There was no difference in weight among the calves under the different treatments at the beginning of the experiment. However, an increase in the dietary NDF levels resulted in a linear decrease or a downward trend in both final BW and weaning BW. Castells et al. (39) concluded that roughage feeding did not affect intestinal filling in calves, as the impact was negligible when forage intake constituted less than 5% of total solid feed intake. Thus, the observed enhancement in production performance in the present study was not caused by intestinal filling. Conversely, Mirzaei et al. (16) suggested that the retention of forage in the rumen, caused by feeding roughage to calves, is the main cause of weight gain. Nemati et al. (21) showed that feeding alfalfa with 34.04% DM increased final BW. It is generally believed that an increase in BW gains from forage feeding is attributed to the increased gastrointestinal tissue weight due to intestinal filling (4, 9, 10, 16, 40). However, our study indicated that final BW decreased with increasing NDF levels, suggesting that gut filling may be a misleading factor when assessing the impact of forage on production performance (16). Nemati et al. (21) also found that feeding calves roughage could induce gut-filling effects, potentially increasing heart and abdominal circumferences. In our study, the body length and total heart girth decreased or tended to decrease linearly with increasing dietary NDF level, with greater measurements observed in the calves fed the 12.85% or 19.91% NDF starter diets at 112 days of age. These findings suggest that starter diets containing less than 26.99% NDF enhance the growth rate and body size in calves.

### Blood metabolites

Plasma BHBA levels serve as indicators of rumen metabolic activity, with BHBA being derived from ruminal butyrate via the rumen wall (41). The plasma BHBA concentrations found in this study were greater than those documented previously (21). Similar blood BHBA concentrations were observed in calves fed either hay or no hay (4). In the present study, the increasing dietary NDF levels linearly increased the blood BHBA concentrations at 70 days of age. We hypothesized that variations in blood BHBA concentrations are primarily due to differences in ruminal metabolic development (42) and calves’ fiber fermentation capacity (43). Khan et al. (4) demonstrated that BHBA concentrations decreased with calf age, contrasting with other studies that reported an increase with age (8, 44), which was not observed in this study. Therefore, further investigation is required in this area.

No difference in the blood glucose concentrations was observed among the treatments in this study, indicating that the calves maintained a similar energy status across the diets (45). Blood glucose concentrations were expected to decrease with calf age, which was consistent with the findings of Khan et al. (4) and Nemati et al. (21), primarily due to a physiological shift from glucose to VFAs as the main energy source. In addition, BUN concentrations increased with age, which could be attributed to higher starter intake. Senevirathne et al. (45) also reported that increased starter consumption elevated plasma urea nitrogen concentrations as the calves aged.

## Conclusion

Under the experimental conditions, increasing dietary NDF concentration in the starter diets linearly decreased the ADG, with higher gains observed at the 12.85 and 19.91% NDF diets during both the pre- and post-weaning periods. The starter intake and TDMI increased quadratically with increasing NDF concentration in the starter diets, peaking at 26.99% NDF. The FE decreased with elevated NDF levels post-weaning, while the 12.85 and 19.91% NDF diets maintained superior efficiency. Moreover, the body length decreased linearly with increasing NDF levels in the starter diets, with the 12.85 and 19.91% NDF diets yielding greater values at 112 days of age. These results suggest that the dietary NDF content below 26.99% may enhance performance and growth in 1- to 3-month-old Holstein calves. Further research is recommended to explore the effects of NDF levels on ruminal fermentation and blood BHBA concentration in calves.

## Data Availability

The original contributions presented in the study are included in the article/supplementary material, further inquiries can be directed to the corresponding author/s.
